# (*E*)-2-(4-Benz­yloxy-2-hy­droxy­benzyl­idene)-*N*-methyl­hydrazinecarbo­thio­amide

**DOI:** 10.1107/S1600536813034892

**Published:** 2014-01-08

**Authors:** N. R. Sajitha, M. Sithambaresan, M. R. Prathapachandra Kurup

**Affiliations:** a Department of Applied Chemistry, Cochin University of Science and Technology, Kochi 682 022, India; b Department of Chemistry, Faculty of Science, Eastern University, Sri Lanka, Chenkalady, Sri Lanka

## Abstract

The mol­ecule of the title compound, C_16_H_17_N_3_O_2_S, adopts an *E* conformation with respect to the azomethine C=N bond. The hydrazinecarbo­thio­amide fragment is close to planar, with a largest deviation from the least-squares plane of 0.079 (2) Å for the hydrazide N atom. This fragment forms a dihedral angle of 9.43 (9)° with the central benzene ring. The benzene rings are inclined to one another by 67.55 (12)°. The mol­ecular conformation is stabilized by an intra­molecular O—H⋯N hydrogen bond involving the azomethine N atom. In the crystal, mol­ecules are linked through weak N—H⋯S and N—H⋯O hydrogen bonds into double ribbons along [010]. The crystal packing also features C—H⋯π inter­actions.

## Related literature   

For catalytic properties of complexes containing thio­semicarbazone ligands, see: Moradi-Shoeili *et al.* (2013 ▶) and for the use of such complexes in imaging and therapy, see: Dilworth & Hueting (2012 ▶). For the synthesis and structure of a closely related compound, see: Nisha *et al.* (2011 ▶). For related structures, see: Seena *et al.* (2006 ▶, 2008 ▶); Jacob & Kurup (2012 ▶); Tarafder *et al.* (2008 ▶).
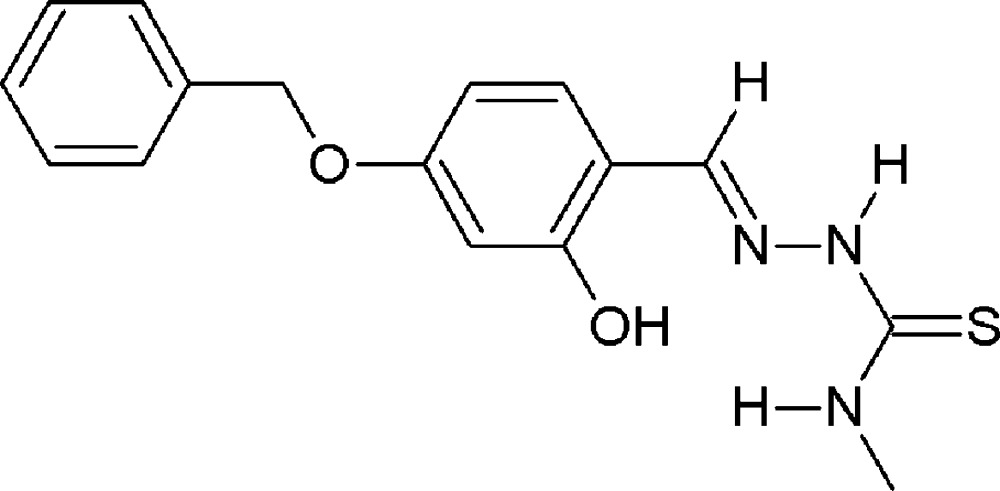


## Experimental   

### 

#### Crystal data  

C_16_H_17_N_3_O_2_S
*M* *_r_* = 315.39
Monoclinic, 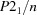

*a* = 17.013 (2) Å
*b* = 5.9474 (10) Å
*c* = 17.542 (4) Å
β = 117.565 (7)°
*V* = 1573.4 (5) Å^3^
*Z* = 4
Mo *K*α radiation
μ = 0.22 mm^−1^
*T* = 296 K
0.50 × 0.40 × 0.35 mm

#### Data collection  

Bruker KAPPA APEXII CCD area-detector diffractometer
Absorption correction: multi-scan (*SADABS*; Bruker, 2007 ▶) *T*_min_ = 0.900, *T*_max_ = 0.928
10845 measured reflections
3827 independent reflections
2577 reflections with *I* > 2σ(*I*)
*R*_int_ = 0.023

#### Refinement  

*R*[*F* ^2^ > 2σ(*F* ^2^)] = 0.043
*wR*(*F* ^2^) = 0.128
*S* = 1.02
3827 reflections
212 parameters
3 restraints
H atoms treated by a mixture of independent and constrained refinement
Δρ_max_ = 0.24 e Å^−3^
Δρ_min_ = −0.24 e Å^−3^

### 

Data collection: *APEX2* (Bruker, 2007 ▶); cell refinement: *SAINT* (Bruker, 2007 ▶); data reduction: *SAINT*; program(s) used to solve structure: *SHELXS97* (Sheldrick, 2008 ▶); program(s) used to refine structure: *SHELXL97* (Sheldrick, 2008 ▶); molecular graphics: *ORTEP-3 for Windows* (Farrugia, 2012 ▶) and *DIAMOND* (Brandenburg, 2010 ▶); software used to prepare material for publication: *SHELXL97* and *publCIF* (Westrip, 2010 ▶).

## Supplementary Material

Crystal structure: contains datablock(s) Global, I. DOI: 10.1107/S1600536813034892/yk2101sup1.cif

Structure factors: contains datablock(s) I. DOI: 10.1107/S1600536813034892/yk2101Isup2.hkl

Supporting information file. DOI: 10.1107/S1600536813034892/yk2101Isup3.cml

CCDC reference:

Additional supporting information:  crystallographic information; 3D view; checkCIF report

## Figures and Tables

**Table 1:** Hydrogen-bond geometry (Å, °) *Cg*2 is the centroid of the C8–C13 ring.

*D*—H⋯*A*	*D*—H	H⋯*A*	*D*⋯*A*	*D*—H⋯*A*
N3—H3′⋯S1^i^	0.87 (1)	2.84 (2)	3.4382 (17)	127 (2)
N2—H2′⋯O2^ii^	0.88 (1)	2.48 (2)	3.094 (2)	127 (2)
N2—H2′⋯S1^iii^	0.88 (1)	2.77 (2)	3.4850 (17)	139 (2)
O2—H2*A*⋯N1	0.84 (1)	2.00 (2)	2.690 (2)	140 (3)
C2—H2⋯*Cg*2^iv^	0.93	2.93	3.6451 (15)	135

Symmetry codes: (i) 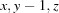; (ii) 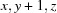; (iii) 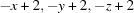; (iv) 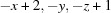.

**Fig. 1.**
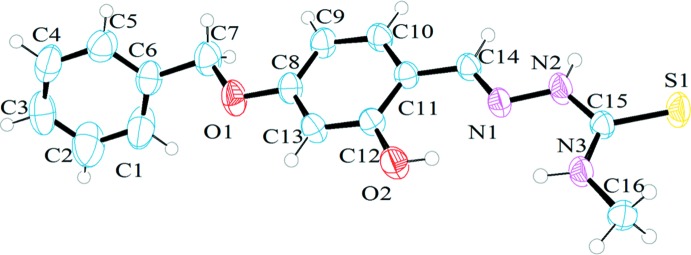
ORTEP diagram of the title compound with 50% probability thermal ellipsoids and atom labelling scheme.

**Fig. 2.**
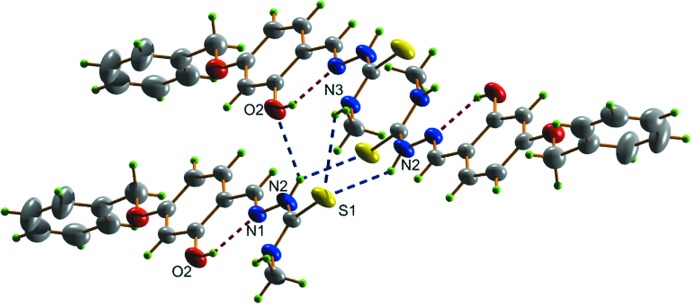
Hydrogen-bond interactions in the title compound.

**Fig. 3.**
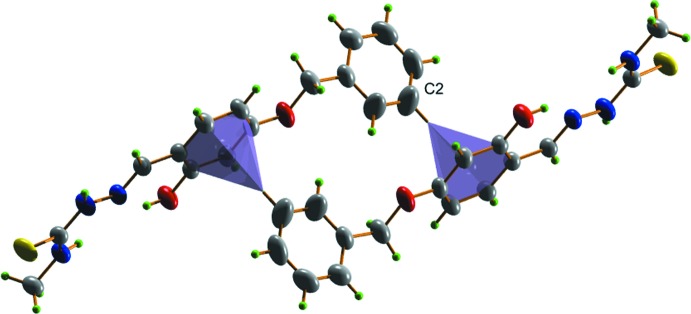
C—H···π interaction in the title compound.

**Fig. 4.**
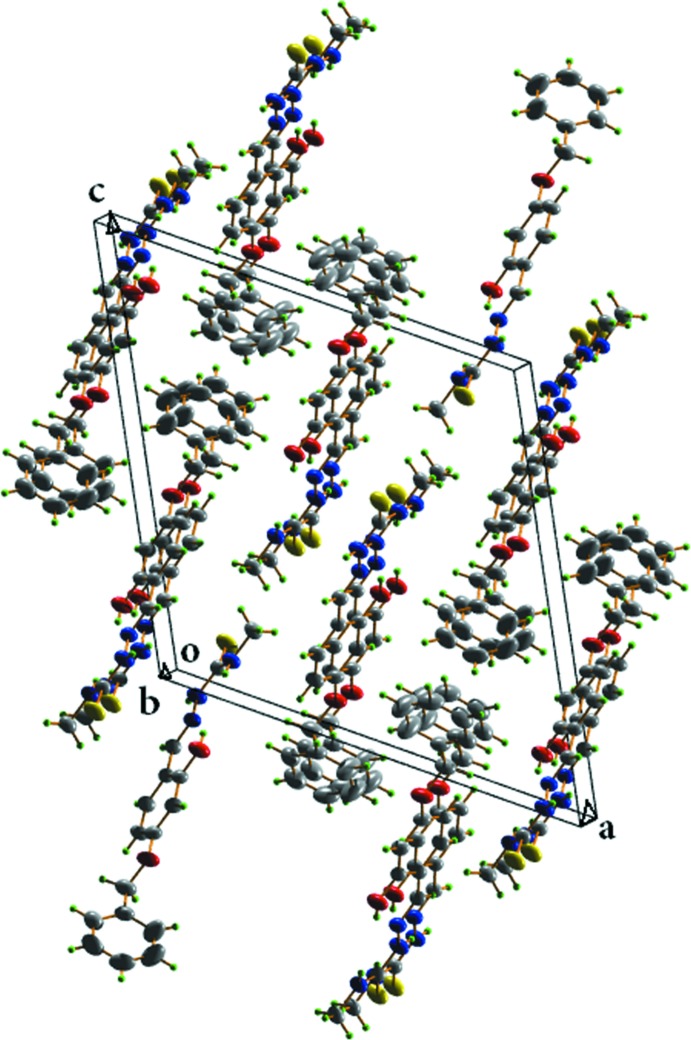
Packing diagram of the title compound along the b axis direction.

## supplementary crystallographic information

### 1. Comment

Hydrazinecarbothioamides are important class of multidentate ligands owing to their application in various fields of medicine and industry. The complexes of these compounds find application as catalysts in organic syntheses (Moradi-Shoeili *et al.*, 2013) and in imaging and therapy in medicine (Dilworth & Hueting, 2012).

The title compound adopts *E* configuration with respect to C14—N1 bond (Fig.1), with C14/N1/N2/C15 torsion angle of 175.19 (17)°. The N1/N2/C15/S1 torsion angle of 170.34 (14)° suggests that the thionyl atom S1 is located *trans* to azomethine nitrogen N1. The C14—N1 bond distance [1.283 (2) Å] is close to that of formal double C═N bond [1.284 (3) Å] (Seena *et al.*, 2006). Similarly the C15—S1 bond distance [1.6837 (18) Å] is also close to that of formal C═S bond [1.685 (3) Å] (Jacob & Kurup, 2012).

The molecule as a whole is non-planar, the two benzene rings are twisted by a dihedral angle of 67.55 (12)°. The hydrazinecarbothiamide fragment N1/N3/C15/S1/C16 is planar with maximum deviation of 0.020 (2) Å for N3, similar to that of salicylaldehyde-*N*(4)-phenylthiosemicarbazone (Seena *et al.*, 2008).

An intramolecular O2—H2A···N1 hydrogen bond found in the molecule with a D···A distance of 2.690 (2) Å forms a six membered ring strengthening the rigidity of the molecule. The molecules are held together through three N—H···S and N—H···O classical intermolecular hydrogen bonds (Fig. 2) with D···A distances of 3.4382 (17), 3.094 (2) and 3.4850 (17) Å. Only one C—H···π interaction (Fig. 3) is present in the molecule with *C*···*Cg* distance of 3.645 (4) Å (Table 1), which also contributes to the arrangement of molecules in the crystal. The molecules are stacked along the *b* axis as shown in Fig. 4.

### 2. Experimental

The title compound was prepared by adapting a reported procedure (Nisha *et al.*, 2011). 4-Methylthiosemicarbazone (0.1050 g, 1 mmol) in 20 ml acetonitrile was acidified with glacial acetic acid and refluxed with 4-benzyloxy-2-hydroxybenzaldehyde (0.2181 g, 1 mmol) in acetonitrile. The resulting solution was kept for one week and the yellow block shaped crystals obtained in 60% yield were separated and analysed.

IR (KBr, \v in cm^-1^): 3344, 3205, 2945, 1555, 1337, 1263, 1222. ^1^H NMR (400 MHz, DMSO-d_6_, δ in p.p.m.): 11.26 (s, 1H), 9.96 (s, 1H), 8.28 (s, 1H), 8.33–8.31 (m, 1H), 7.83–7.81 (m, 1H), 7.46–7.40 (m,5*H*), 7.38–7.32 (m,2*H*), 5.09 (s,2*H*), 3.00–3.01 (d,3*H*).

### 3. Refinement

All H atoms on C were placed in calculated positions, guided by difference map, with C—H bond distances of 0.93 Å. H atoms were assigned *U*_iso_(H) values of 1.2Ueq (carrier). H atoms of N2—H2' and N3—H3' bonds were located from difference maps and the bond distances are restrained to 0.88±0.01 Å. Omitted owing to bad disagreement was (-1 0 1). H atom of O2—H2A bond was located from difference maps and the bond distance is restrained to 0.84±0.01 Å.

### Crystal data

**Table:**

C_16_H_17_N_3_O_2_S	*F*(000) = 664
*M**_r_* = 315.39	*D*_x_ = 1.331 Mg m^−^^3^
Monoclinic, *P*2_1_/*n*	Mo *K*α radiation, λ = 0.71073 Å
Hall symbol: -P 2yn	Cell parameters from 740 reflections
*a* = 17.013 (2) Å	θ = 3.7–28.0°
*b* = 5.9474 (10) Å	µ = 0.22 mm^−^^1^
*c* = 17.542 (4) Å	*T* = 296 K
β = 117.565 (7)°	Block, yellow
*V* = 1573.4 (5) Å^3^	0.50 × 0.40 × 0.35 mm
*Z* = 4	

### Data collection

**Table:**

Bruker KAPPA APEXII CCD area-detector diffractometer	3827 independent reflections
Radiation source: fine-focus sealed tube	2577 reflections with *I* > 2σ(*I*)
Graphite monochromator	*R*_int_ = 0.023
ω and φ scan	θ_max_ = 28.2°, θ_min_ = 2.3°
Absorption correction: multi-scan (*SADABS*; Bruker, 2007)	*h* = −22→22
*T*_min_ = 0.900, *T*_max_ = 0.928	*k* = −7→4
10845 measured reflections	*l* = −22→23

### Refinement

**Table:**

Refinement on *F*^2^	Primary atom site location: structure-invariant direct methods
Least-squares matrix: full	Secondary atom site location: difference Fourier map
*R*[*F*^2^ > 2σ(*F*^2^)] = 0.043	Hydrogen site location: inferred from neighbouring sites
*wR*(*F*^2^) = 0.128	H atoms treated by a mixture of independent and constrained refinement
*S* = 1.02	*w* = 1/[σ^2^(*F*_o_^2^) + (0.0541*P*)^2^ + 0.5741*P*] where *P* = (*F*_o_^2^ + 2*F*_c_^2^)/3
3827 reflections	(Δ/σ)_max_ = 0.001
212 parameters	Δρ_max_ = 0.24 e Å^−^^3^
3 restraints	Δρ_min_ = −0.24 e Å^−^^3^

### Special details

**Table:**

Geometry. All e.s.d.'s (except the e.s.d. in the dihedral angle between two l.s. planes) are estimated using the full covariance matrix. The cell e.s.d.'s are taken into account individually in the estimation of e.s.d.'s in distances, angles and torsion angles; correlations between e.s.d.'s in cell parameters are only used when they are defined by crystal symmetry. An approximate (isotropic) treatment of cell e.s.d.'s is used for estimating e.s.d.'s involving l.s. planes.
Refinement. Refinement of *F*^2^ against ALL reflections. The weighted *R*-factor *wR* and goodness of fit *S* are based on *F*^2^, conventional *R*-factors *R* are based on *F*, with *F* set to zero for negative *F*^2^. The threshold expression of *F*^2^ > σ(*F*^2^) is used only for calculating *R*-factors(gt) *etc*. and is not relevant to the choice of reflections for refinement. *R*-factors based on *F*^2^ are statistically about twice as large as those based on *F*, and *R*- factors based on ALL data will be even larger.

### Fractional atomic coordinates and isotropic or equivalent isotropic displacement parameters (Å^2^)

**Table:**

	*x*	*y*	*z*	*U*_iso_*/*U*_eq_	
S1	1.13656 (4)	0.95873 (8)	1.10134 (3)	0.05333 (18)	
O1	0.90536 (10)	−0.0765 (2)	0.57207 (9)	0.0527 (4)	
O2	1.06521 (10)	0.1191 (2)	0.86083 (9)	0.0519 (4)	
N1	1.04073 (10)	0.5297 (2)	0.91066 (9)	0.0370 (3)	
N2	1.05299 (11)	0.7111 (3)	0.96424 (10)	0.0440 (4)	
N3	1.16019 (10)	0.5258 (2)	1.07980 (10)	0.0396 (4)	
C1	0.9035 (2)	−0.2867 (5)	0.41772 (19)	0.0874 (10)	
H1	0.9544	−0.1986	0.4424	0.105*	
C2	0.8998 (2)	−0.4661 (6)	0.3667 (2)	0.0962 (11)	
H2	0.9481	−0.4992	0.3574	0.115*	
C3	0.8253 (2)	−0.5953 (4)	0.32960 (16)	0.0735 (8)	
H3	0.8226	−0.7156	0.2946	0.088*	
C4	0.75516 (18)	−0.5479 (4)	0.34383 (16)	0.0676 (7)	
H4	0.7044	−0.6365	0.3187	0.081*	
C5	0.75889 (16)	−0.3694 (4)	0.39528 (14)	0.0588 (6)	
H5	0.7106	−0.3384	0.4049	0.071*	
C6	0.83341 (15)	−0.2365 (4)	0.43258 (13)	0.0511 (5)	
C7	0.83740 (16)	−0.0377 (4)	0.48654 (14)	0.0583 (6)	
H7A	0.8504	0.0973	0.4635	0.070*	
H7B	0.7808	−0.0176	0.4865	0.070*	
C8	0.92092 (13)	0.0866 (3)	0.63194 (11)	0.0398 (4)	
C9	0.87738 (13)	0.2932 (3)	0.61485 (12)	0.0435 (4)	
H9	0.8347	0.3299	0.5595	0.052*	
C10	0.89893 (13)	0.4411 (3)	0.68168 (12)	0.0411 (4)	
H10	0.8704	0.5798	0.6703	0.049*	
C11	0.96147 (12)	0.3927 (3)	0.76555 (11)	0.0348 (4)	
C12	1.00432 (12)	0.1827 (3)	0.78095 (11)	0.0367 (4)	
C13	0.98461 (13)	0.0341 (3)	0.71415 (12)	0.0409 (4)	
H13	1.0145	−0.1025	0.7246	0.049*	
C14	0.98145 (12)	0.5582 (3)	0.83231 (11)	0.0368 (4)	
H14	0.9498	0.6924	0.8178	0.044*	
C15	1.11670 (12)	0.7152 (3)	1.04658 (11)	0.0353 (4)	
C16	1.22781 (13)	0.5050 (3)	1.16849 (12)	0.0447 (5)	
H16A	1.2759	0.6053	1.1788	0.067*	
H16B	1.2493	0.3531	1.1793	0.067*	
H16C	1.2029	0.5428	1.2060	0.067*	
H3'	1.1452 (13)	0.405 (2)	1.0482 (11)	0.044 (6)*	
H2'	1.0253 (14)	0.838 (3)	0.9419 (14)	0.073 (8)*	
H2A	1.0769 (19)	0.218 (4)	0.8988 (14)	0.098 (10)*	

### Atomic displacement parameters (Å^2^)

**Table:**

	*U*^11^	*U*^22^	*U*^33^	*U*^12^	*U*^13^	*U*^23^
S1	0.0635 (4)	0.0282 (2)	0.0462 (3)	0.0024 (2)	0.0066 (2)	−0.0091 (2)
O1	0.0752 (10)	0.0424 (8)	0.0337 (7)	0.0006 (7)	0.0193 (7)	−0.0072 (6)
O2	0.0643 (10)	0.0430 (8)	0.0345 (8)	0.0158 (7)	0.0110 (7)	0.0000 (6)
N1	0.0466 (9)	0.0283 (7)	0.0334 (8)	0.0010 (6)	0.0161 (7)	−0.0033 (6)
N2	0.0573 (10)	0.0268 (8)	0.0358 (9)	0.0080 (7)	0.0111 (8)	−0.0034 (6)
N3	0.0471 (9)	0.0278 (8)	0.0353 (8)	0.0027 (6)	0.0119 (7)	−0.0034 (6)
C1	0.090 (2)	0.096 (2)	0.100 (2)	−0.0458 (17)	0.0645 (19)	−0.0536 (18)
C2	0.108 (2)	0.107 (2)	0.108 (3)	−0.0363 (19)	0.079 (2)	−0.052 (2)
C3	0.110 (2)	0.0591 (15)	0.0524 (15)	−0.0159 (14)	0.0386 (15)	−0.0195 (12)
C4	0.0705 (16)	0.0540 (14)	0.0583 (15)	−0.0166 (12)	0.0129 (13)	−0.0088 (11)
C5	0.0554 (13)	0.0597 (14)	0.0501 (13)	−0.0030 (11)	0.0149 (11)	−0.0017 (11)
C6	0.0635 (14)	0.0533 (12)	0.0346 (11)	−0.0098 (10)	0.0211 (10)	−0.0077 (9)
C7	0.0695 (15)	0.0569 (13)	0.0398 (12)	−0.0025 (11)	0.0179 (11)	−0.0092 (10)
C8	0.0516 (11)	0.0378 (10)	0.0325 (9)	−0.0053 (8)	0.0215 (9)	−0.0030 (7)
C9	0.0510 (12)	0.0397 (10)	0.0307 (9)	0.0012 (8)	0.0114 (9)	0.0021 (8)
C10	0.0474 (11)	0.0326 (9)	0.0386 (10)	0.0050 (8)	0.0159 (9)	0.0027 (7)
C11	0.0401 (10)	0.0309 (8)	0.0333 (9)	0.0003 (7)	0.0169 (8)	0.0006 (7)
C12	0.0429 (10)	0.0350 (9)	0.0320 (9)	0.0031 (7)	0.0172 (8)	0.0021 (7)
C13	0.0523 (11)	0.0326 (9)	0.0390 (10)	0.0051 (8)	0.0220 (9)	0.0003 (7)
C14	0.0427 (10)	0.0299 (9)	0.0348 (9)	0.0033 (7)	0.0154 (8)	0.0003 (7)
C15	0.0394 (10)	0.0276 (8)	0.0356 (9)	−0.0003 (7)	0.0146 (8)	−0.0018 (7)
C16	0.0441 (10)	0.0370 (10)	0.0411 (11)	0.0046 (8)	0.0096 (9)	0.0009 (8)

### Geometric parameters (Å, º)

**Table:**

S1—C15	1.6837 (18)	C4—H4	0.9300
O1—C8	1.362 (2)	C5—C6	1.376 (3)
O1—C7	1.427 (3)	C5—H5	0.9300
O2—C12	1.355 (2)	C6—C7	1.496 (3)
O2—H2A	0.840 (10)	C7—H7A	0.9700
N1—C14	1.285 (2)	C7—H7B	0.9700
N1—N2	1.382 (2)	C8—C13	1.380 (3)
N2—C15	1.346 (2)	C8—C9	1.394 (3)
N2—H2'	0.879 (10)	C9—C10	1.373 (3)
N3—C15	1.325 (2)	C9—H9	0.9300
N3—C16	1.450 (2)	C10—C11	1.390 (2)
N3—H3'	0.871 (9)	C10—H10	0.9300
C1—C6	1.367 (3)	C11—C12	1.408 (2)
C1—C2	1.375 (4)	C11—C14	1.445 (2)
C1—H1	0.9300	C12—C13	1.380 (2)
C2—C3	1.362 (4)	C13—H13	0.9300
C2—H2	0.9300	C14—H14	0.9300
C3—C4	1.357 (4)	C16—H16A	0.9600
C3—H3	0.9300	C16—H16B	0.9600
C4—C5	1.376 (3)	C16—H16C	0.9600
			
C8—O1—C7	117.99 (16)	H7A—C7—H7B	108.4
C12—O2—H2A	114 (2)	O1—C8—C13	115.02 (16)
C14—N1—N2	114.92 (14)	O1—C8—C9	124.57 (17)
C15—N2—N1	122.48 (14)	C13—C8—C9	120.41 (17)
C15—N2—H2'	117.6 (17)	C10—C9—C8	118.43 (17)
N1—N2—H2'	119.0 (16)	C10—C9—H9	120.8
C15—N3—C16	123.28 (15)	C8—C9—H9	120.8
C15—N3—H3'	118.8 (14)	C9—C10—C11	122.83 (17)
C16—N3—H3'	117.7 (14)	C9—C10—H10	118.6
C6—C1—C2	120.8 (2)	C11—C10—H10	118.6
C6—C1—H1	119.6	C10—C11—C12	117.51 (16)
C2—C1—H1	119.6	C10—C11—C14	119.64 (16)
C3—C2—C1	120.1 (3)	C12—C11—C14	122.83 (16)
C3—C2—H2	120.0	O2—C12—C13	117.94 (16)
C1—C2—H2	120.0	O2—C12—C11	121.75 (16)
C4—C3—C2	119.9 (2)	C13—C12—C11	120.31 (17)
C4—C3—H3	120.1	C12—C13—C8	120.48 (17)
C2—C3—H3	120.1	C12—C13—H13	119.8
C3—C4—C5	120.2 (2)	C8—C13—H13	119.8
C3—C4—H4	119.9	N1—C14—C11	123.42 (16)
C5—C4—H4	119.9	N1—C14—H14	118.3
C4—C5—C6	120.5 (2)	C11—C14—H14	118.3
C4—C5—H5	119.7	N3—C15—N2	117.71 (15)
C6—C5—H5	119.7	N3—C15—S1	123.80 (14)
C1—C6—C5	118.5 (2)	N2—C15—S1	118.49 (13)
C1—C6—C7	120.3 (2)	N3—C16—H16A	109.5
C5—C6—C7	121.1 (2)	N3—C16—H16B	109.5
O1—C7—C6	108.36 (18)	H16A—C16—H16B	109.5
O1—C7—H7A	110.0	N3—C16—H16C	109.5
C6—C7—H7A	110.0	H16A—C16—H16C	109.5
O1—C7—H7B	110.0	H16B—C16—H16C	109.5
C6—C7—H7B	110.0		
			
C14—N1—N2—C15	−175.19 (17)	C9—C10—C11—C12	0.6 (3)
C6—C1—C2—C3	0.5 (5)	C9—C10—C11—C14	179.20 (17)
C1—C2—C3—C4	−0.6 (5)	C10—C11—C12—O2	−178.94 (17)
C2—C3—C4—C5	0.3 (4)	C14—C11—C12—O2	2.5 (3)
C3—C4—C5—C6	0.3 (4)	C10—C11—C12—C13	0.7 (3)
C2—C1—C6—C5	0.0 (5)	C14—C11—C12—C13	−177.86 (16)
C2—C1—C6—C7	−178.5 (3)	O2—C12—C13—C8	177.77 (17)
C4—C5—C6—C1	−0.4 (4)	C11—C12—C13—C8	−1.9 (3)
C4—C5—C6—C7	178.2 (2)	O1—C8—C13—C12	−177.96 (16)
C8—O1—C7—C6	179.20 (17)	C9—C8—C13—C12	1.8 (3)
C1—C6—C7—O1	−64.9 (3)	N2—N1—C14—C11	177.22 (16)
C5—C6—C7—O1	116.6 (2)	C10—C11—C14—N1	−177.27 (18)
C7—O1—C8—C13	177.13 (18)	C12—C11—C14—N1	1.2 (3)
C7—O1—C8—C9	−2.6 (3)	C16—N3—C15—N2	−177.52 (17)
O1—C8—C9—C10	179.22 (17)	C16—N3—C15—S1	2.4 (3)
C13—C8—C9—C10	−0.5 (3)	N1—N2—C15—N3	−9.7 (3)
C8—C9—C10—C11	−0.7 (3)	N1—N2—C15—S1	170.34 (14)

### Hydrogen-bond geometry (Å, º)

Cg2 is the centroid of the C8–C13 ring.

**Table:**

*D*—H···*A*	*D*—H	H···*A*	*D*···*A*	*D*—H···*A*
N3—H3′···S1^i^	0.87 (1)	2.84 (2)	3.4382 (17)	127 (2)
N2—H2′···O2^ii^	0.88 (1)	2.48 (2)	3.094 (2)	127 (2)
N2—H2′···S1^iii^	0.88 (1)	2.77 (2)	3.4850 (17)	139 (2)
O2—H2*A*···N1	0.84 (1)	2.00 (2)	2.690 (2)	140 (3)
C2—H2···*Cg*2^iv^	0.93	2.93	3.6451 (15)	135

Symmetry codes: (i) *x*, *y*−1, *z*; (ii) *x*, *y*+1, *z*; (iii) −*x*+2, −*y*+2, −*z*+2; (iv) −*x*+2, −*y*, −*z*+1.

## References

[bb1] Brandenburg, K. (2010). *DIAMOND* Crystal Impact GbR, Bonn, Germany.

[bb2] Bruker (2007). *APEX2*, *SAINT* and *SADABS* Bruker AXS Inc., Madison, Wisconsin, USA.

[bb3] Dilworth, J. R. & Hueting, R. (2012). *Inorg. Chim. Acta*, **389**, 3–15.

[bb4] Farrugia, L. J. (2012). *J. Appl. Cryst.* **45**, 849–854.

[bb5] Jacob, J. M. & Kurup, M. R. P. (2012). *Acta Cryst.* E**68**, o836–o837. 10.1107/S1600536812007039 PMC3297895 22412698

[bb6] Moradi-Shoeili, Z., Boghaei, D. M., Amini, M. & Notash, B. (2013). *Inorg. Chem. Commun.* **27**, 26–30.

[bb7] Nisha, K., Sithambaresan, M. & Kurup, M. R. P. (2011). *Acta Cryst.* E**67**, o3420. 10.1107/S1600536811049658 PMC3239056 22199904

[bb8] Seena, E. B., BessyRaj, B. N., Kurup, M. R. P. & Suresh, E. (2006). *J. Chem. Crystallogr.* **36**, 189–193.

[bb9] Seena, E. B., Kurup, M. R. P. & Suresh, E. (2008). *J. Chem. Crystallogr.* **38**, 93–96.

[bb10] Sheldrick, G. M. (2008). *Acta Cryst.* A**64**, 112–122. 10.1107/S0108767307043930 18156677

[bb11] Tarafder, M. T. H., Islam, M. A. A. A. A., Crouse, K. A., Chantrapromma, S. & Fun, H.-K. (2008). *Acta Cryst.* E**64**, o988–o989. 10.1107/S1600536808012671 PMC2961576 21202714

[bb12] Westrip, S. P. (2010). *J. Appl. Cryst.* **43**, 920–925.

